# Gender Disparities in Latent Tuberculosis Infection in High-Risk Individuals: A Cross-Sectional Study

**DOI:** 10.1371/journal.pone.0110104

**Published:** 2014-11-04

**Authors:** Wen-Ying Ting, Shiang-Fen Huang, Ming-Che Lee, Yung-Yang Lin, Yu-Chin Lee, Jia-Yih Feng, Wei-Juin Su

**Affiliations:** 1 Department of Chest Medicine, Taipei Veterans General Hospital, Taipei, Taiwan, R.O.C.; 2 Division of Infectious Disease, Department of Internal Medicine, Taipei Veterans General Hospital, Taipei, Taiwan, R.O.C.; 3 School of Medicine, National Yang-Ming University, Taipei, Taiwan, R.O.C.; 4 Institute of Clinical Medicine and Institute of Brain Science, National Yang-Ming University, Taipei, Taiwan, R.O.C.; 5 Laboratory of Neurophysiology and Department of Neurology, Taipei Veterans General Hospital, Taipei, Taiwan, R.O.C.; 6 Institute of Clinical Medicine, School of Medicine, National Yang-Ming University, Taipei, Taiwan, R.O.C.; University of Pennsylvania School of Medicine, United States of America

## Abstract

Male predominance in active tuberculosis (TB) is widely-reported globally. Gender inequalities in socio-cultural status are frequently regarded as contributing factors for disparities in sex in active TB. The disparities of sex in the prevalence of latent TB infection (LTBI) are less frequently investigated and deserve clarification. In this cross-sectional study conducted in a TB endemic area, we enrolled patients at high-risk for LTBI and progression from LTBI to active TB from 2011 to 2012. Diagnosis of LTBI was made by QuantiFERON-TB Gold In-Tube (QFT-GIT). Differences in sex in terms of prevalence of LTBI and clinical predictors for LTBI were investigated. Associations among age, smoking status, and sex disparities in LTBI were also analyzed. A total of 1018 high-risk individuals with definite QFT-GIT results were included for analysis, including 534 males and 484 females. The proportion of LTBI was significantly higher in males than in females (32.6% vs. 25.2%, *p* = 0.010). Differences in the proportion of LTBI between sexes were most prominent in older patients (age ≥55 years). In multivariate analysis, independent clinical factors associated with LTBI were age (*p* = 0.014), smoking (*p* = 0.048), and fibro-calcified lesions on chest radiogram (*p* = 0.009). Male sex was not an independent factor for LTBI (*p* = 0.88). When stratifying patients according to the smoking status, the proportion of LTBI remained comparable between sexes among smokers and non-smokers. In conclusion, although the proportion of LTBI is higher in men, there is no significant disparity in terms of sex in LTBI among high-risk individuals after adjusting for age, smoking status, and other clinical factors.

## Introduction

Tuberculosis (TB) is caused by *Mycobacterium tuberculosis* (MTB) and is one of the deadliest infectious diseases worldwide. Despite recent progress in molecular diagnosis and effective medications, its morbidity and mortality remain high. The World Health Organization (WHO) reported that 8.7 million people developed active TB in 2011 and 1.4 million people died from it [1]. Meanwhile, one-third of the world's population is estimated to be infected by MTB. Latent TB infection (LTBI) is defined by evidence of immunological responses by *Mycobacterium tuberculosis* (MTB) proteins in the absence of clinical symptoms/signs of active diseases [2]. An estimated 30% of the people exposed to MTB will have evidence of LTBI by tuberculin skin test [3]. By definition, LTBI cases do not have clinical or radiographic evidence of the disease and will not cause transmission. However, a significant proportion of patients with LTBI will progress to active disease and it is preventable by effective treatment. Therefore, identifying and sterilizing latently infected individuals, especially those at high risk, are of paramount importance for eliminating TB [4].

Sex differences in the epidemiology and treatment outcomes of active TB are remarkable and have been well-described in previous reports [5]–[8]. In general, men are more likely to be diagnosed with active TB than women, with a male-to-female ratio of 2∶1 to 3∶1 globally [1]. Males with active TB also have worse outcomes, including delayed sputum conversion, higher reactivation rate, and higher mortality rate, compared to females [9]–[11]. The impact of smoking, inequalities in socio-economic status, differences in medical accessibility, and sex hormone-related differences in immunity are reported as possible causes for the disparities in sexes in active TB [12]–[15]. However, the exact mechanisms remain unclear.

Compared to numerous reports on active TB, disparities between sexes in LTBI are less frequently analyzed and have inconsistent findings. Male sex has been identified as an independent risk factor associated with LTBI in some studies [16]–[18], but several studies also report insignificant correlation between sex and LTBI [19], [20]. Most studies have focused on specific populations with relatively few case numbers. Important clinical characteristic profiles, especially smoking status, are also lacking. Given the uncertainty of the mechanisms related to sex disparities in active TB, analyzing sex differences in LTBI from an active case-finding setting will be helpful in elucidating the issue. The present study aimed to investigate differences between sexes in LTBI among high-risk individuals. The associated clinical factors, especially age and smoking habits, and their impact on sex disparities, were also evaluated.

## Materials and Methods

### Study Design and Settings

This cross-sectional study was conducted at Taipei Veterans General Hospital, a 3000-bed tertiary medical center in Taiwan where more than 450 active TB cases are diagnosed each year. As a TB endemic area with moderate TB burden, Taiwan had 12,634 newly diagnosed TB cases in 2011, with an annual incidence of 54.5 cases/100,000 population [21]. From 2005 to 2010, there was a 23.3% decrease in number of TB cases and 24.8% decrease in incidence rate.

### Patients and Data Collection

From January 2011 to December 2012, in-patients and out-patients who were considered at risk for LTBI and progression to active TB disease were eligible for enrolledment. These high-risk individuals included people with active TB contact, health care workers, and patients with malignancy, end-stage renal disease, liver cirrhosis, post-organ transplantation, autoimmune diseases, and fibro-calcified lesions suggestive of prior TB on chest radiogram [22].

Patients who were under anti-TB treatment were excluded, as well as those who were diagnosed with active TB (based on the TB registration database of the Centers for Disease Control, Taiwan) within two months of enrollment. The other exclusion criteria included patients younger than 20 years of age, pregnant women, and those with a history of previous anti-TB treatment.

Demographic profiles (age, sex, and co-morbidities) and clinical characteristics (TB contact history, BCG scars, and smoking habit) were obtained by enrollment interviews and medical records. Smoking habit was defined as smoked at least one cigarette a day for at least one year. Body mass index (BMI) was calculated on the day of enrollment. Chest radiographs were taken on enrollment and read by a chest physician who was blinded to the LTBI testing results. Fibro-calcified lesions suggestive of previous TB infection were recorded. The hospital's Institutional Review Board approved this study and all of the patients or their authorized representative(s) provided written informed consent before enrollment.

### Diagnosis of LTBI

The diagnosis of LTBI was determined by interferon-γ release assays, which was performed by the QuantiFERON-TB Gold In-Tube (QFT-GIT; QFT-GIT; Qiagen, Germany) according to the manufacturer's instructions. Peripheral blood (3–5 ml) was obtained from the patients on the day of enrollment. Blood was collected in three special tubes: one coated with MTB-specific peptides (TB antigen), one coated with phytohemaglutinin (mitogen) as a positive control, and one without antigen coating as a negative control (Nil).

Within 8 hours of blood sampling, the tubes were incubated for 16–24 hours at 37°C, centrifuged, and stored at 4°C until assay. The plasma interferon-γ concentration was measured by QFT-GIT enzyme-linked immuno-sorbent assay (ELISA). The test results were determined as negative, intermediate, or positive (cut-off at 0.35 IU/ml) according to the manufacturer's software.

### Statistical Analysis

Statistical analysis was performed using the SPSS version 17.0 software (SPSS, Inc., Chicago, IL, USA). Continuous variables such as age and BMI between sub-groups were compared by Mann-Whitney U tests, while categorical variables were compared using Pearson's chi-square or Fisher's exact tests, as appropriate. Binary logistic regression analysis with stepwise selection was performed to determine the independent variables associated with LTBI in the overall study population and by male and female populations. Crude and adjusted odds ratios (ORs) with their 95% confidence intervals (CI) were presented. A *p*<0.1 in the univariate analysis was required for a variable to be entered into the multivariate model. Patients with indeterminate QFT-GIT results were excluded from the analysis. All tests were two-tailed and *p*<0.05 was considered statistically significant.

## Results

### Study Population

**Figure 1:**
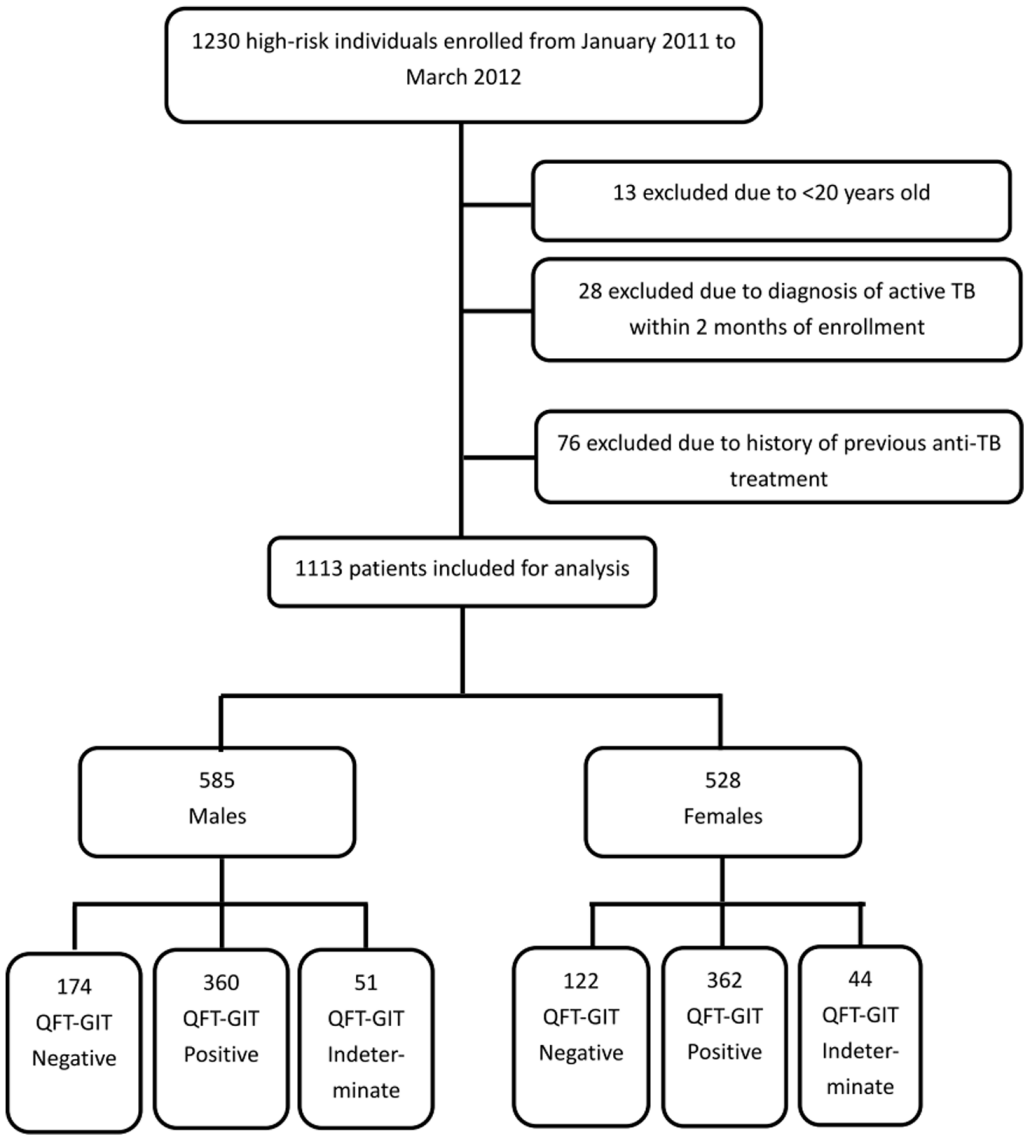
Study profile demonstrating the number of cases and reasons for exclusion.

From January 2011 to December 2012, 1230 high-risk in-patients and out-patients were eligible for recruitment. After a process of exclusion (Figure 1), 1113 patients were enrolled, including 296 (26.6%) who were QFT-GIT positive, 722 (64.9%) who were QFT-GIT negative, and 95 (8.5%) with indeterminate QFT-GIT results. Ultimately, 1018 high-risk individuals composed of 534 (52.5%) males and 484 (47.5%) females with determinate QFT-GIT results were included for analysis. Among them, 152 (14.9%) had recent contact history with active TB cases, 42 (4.1%) were health care workers, 147 (14.4%) had diabetes mellitus, 351 (34.5%) had malignancies, 22 (2.2%) had end-stage renal disease, 44 (4.3%) had liver cirrhosis, 12 (1.2%) had history of organ transplantations, 140 (13.8%) had some autoimmune diseases, and 216 (21.2%) had fibro-calcified lesions on chest radiograms.

Compared to female patients, males were older (*p* = 0.001), more likely to have a smoking habit (*p*<0.001), had COPD (*p*<0.001), had some malignancies (*p*<0.001), and had fibro-calcified lesions on chest radiograms (*p* = 0.012) (Table 1). Males were less likely to have TB contact history (*p*<0.001), be health care workers (*p*<0.001), and have autoimmune disorders (*p*<0.001). The proportion of LTBI in male and females were 32.6% and 25.2% respectively, with statistically significant difference (*p* = 0.010).

**Table 1:** Demographic characteristic between sexes with and without latent TB infection in high-risk individuals^a^^,^ b.

	Males n = 534	Females n = 484	*p* value	Males, n = 534		*p* value	Females, n = 484		*p* value
				With LTBI n = 174	Without LTBI n = 360		With LTBI n = 122	Without LTBI n = 362	
Mean Age (SD)^c^	60.7 (18.9)	57.1 (16.9)	<0.001	65.7 (15.5)	58.3 (19.9)	<0.001	59.8 (15.0)	56.1 (17.4)	0.05
BMI^c^	23.0 (4.3)	22.7 (4.0)	0.015	22.9 (4.0)	23.1 (4.5)	0.84	23.0 (4.0)	22.5 (4.0)	0.36
TB contact history	57 (10.7%)	95 (19.6%)	<0.001	13 (7.5%)	44 (12.2%)	0.10	30 (24.6)	65 (18%)	0.11
Smoking habit	300 (56.2%)	16 (3.3%)	<0.001	118 (67.8%)	182 (50.6%)	<0.001	3 (2.5%)	13 (3.6%)	0.77 d
BCG vaccination	337 (63.1%)	333 (68.8%)	0.06	93 (53.4%)	244 (67.8%)	0.001	79 (64.8%)	254 (70.2%)	0.27
Health care worker	10 (1.9%)	32 (6.6%)	<0.001	4 (2.3%)	6 (1.7%)	0.61^d^	11 (9%)	21 (5.8%)	0.22
Co-morbidity									
Diabetes	80 (15%)	67 (13.8%)	0.61	32 (18.4%)	48 (13.3%)	0.13	16 (13.1%)	51 (14.1%)	0.79
COPD	33 (6.2%)	0	<0.001	18 (10.3%)	15 (4.2%)	0.005	0	0	
Malignancy	243 (45.5%)	108 (22.3%)	<0.001	86 (49.4%)	157 (43.6%)	0.21	20 (16.4%)	88 (24.3%)	0.07
Renal insufficiency	14 (2.6%)	8 (1.7%)	0.29	4 (2.3%)	10 (2.8%)	0.75 d	3 (2.5%)	5 (1.4%)	0.42 d
HIV positive	0	0		0	0		0	0	
Post gastrectomy	14 (2.6%)	5 (1%)	0.06	6 (3.4%)	8 (2.2%)	0.40 d	4 (3.3%)	1 (0.3%)	0.016 d
Liver cirrhosis	28 (5.2%)	16 (3.3%)	0.13	10 (5.7%)	18 (5%)	0.72	1 (1.1%)	15 (4.1%)	0.08 d
Autoimmune disorders	41 (7.7%)	99 (20.4%)	<0.001	12 (6.9%)	29 (8.1%)	0.64	22 (18%)	77 (21.3%)	0.65
Organ transplantation	7 (1.3%)	5 (1%)	0.68	0	7 (1.9%)	0.10 d	0	5 (1.4%)	0.34 d
Fibro-calcified lesions on chest radiogram	128 (24%)	85 (17.6%)	0.012	59 (33.9%)	69 (19.2%)	<0.001	27 (22.1%)	59 (16.3%)	0.15
Latent TB infection	174 (32.6%)	122 (25.2%)	0.010						

a Data are presented as mean ± SD or n (%), unless otherwise stated.

b Only patients with determinate QFT-GIT test results were included for analysis.

c Analyses were performed by Mann-Whitney U test.

d Analyses were performed by Fisher's exact test.

BCG, bacille Calmette-Guerin; BMI, body mass index; COPD, chronic obstructive pulmonary disorder; HIV, human immunodeficiency virus; TB, tuberculosis.

### LTBI between Sexes

The demographic characteristics of individuals with and without LTBI between sexes (Table 1) revealed that in males, patients with LTBI were older (*p*<0.001), more likely to have a smoking habit (*p*<0.001), had COPD (*p* = 0.005), had fibro-calcified lesions on chest radigogram (*p*<0.001), and less likely to have BCG vaccination history (*p* = 0.001) compared with those without LTBI. In females, patients with LTBI were more likely to have a history of gastrectomy (*p* = 0.016).

**Figure 2:**
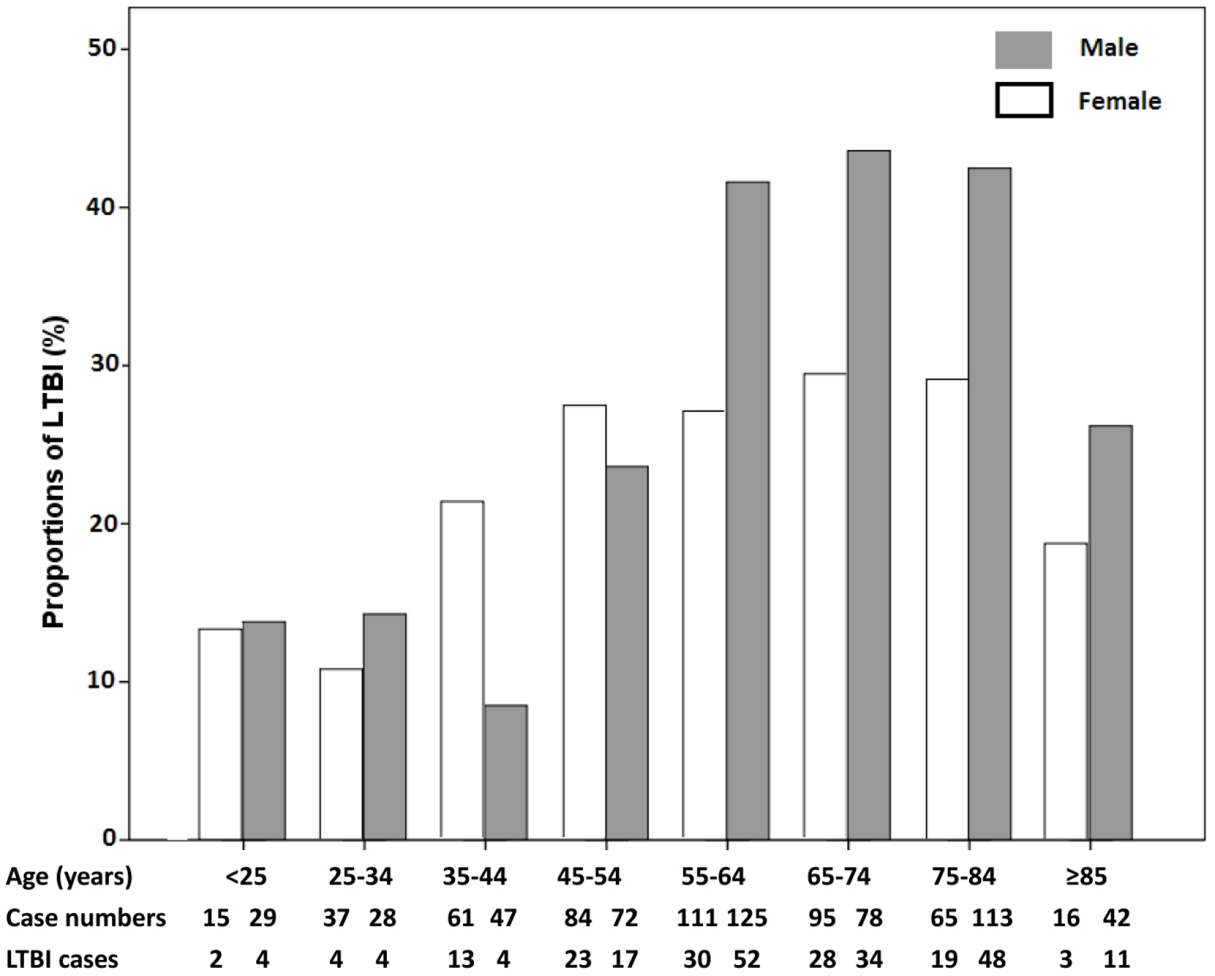
Proportions of latent TB infection between sexes in various age groups.

Because older age was associated with LTBI in both males and females, the proportion of LTBI between sexes in various age groups was analyzed. The proportion of LTBI in males dramatically increased with increasing age, especially in those older than 55 years old (Figure 2). The proportion of LTBI in females also increased with increasing age but the differences were less remarkable. Sex disparities in LTBI were most prominent in patients with age ranged of 55–84 years. The proportion of LTBI declined in the extremely old population, i.e. >85 years old, in both sexes.

### Clinical Factors Associated with LTBI

In multivariate analysis (Table 2), the independent clinical factors associated with LTBI in the overall population included older age (OR 1.013, 95% CI 1.003–1.023; *p* = 0.014), smoking habit (OR 1.46, 95% CI 1.003–2.115; p = 0.048), and fibro-calcified lesions on chest radiogram(OR 1.56, 95% CI 1.12–2.17; *p* = 0.009). Male sex was not a significant factor (OR 1.03, 95% CI 0.72–1.46; *p* = 0.88).

**Table 2:** Univariate and multivariate analysis of clinical factors associated with latent TB infection in high-risk individuals^a^.

	Univariate analysis		Multivariate analysis	
	OR (95% CI)	*p* value	OR (95% CI)	*p* value
Age	1.02 (1.01–1.03)	<0.001	1.013 (1.00–1.02)	0.014
Male sex	1.43 (1.09–1.89)	0.010	1.03 (0.72–1.46)	0.88
BCG vaccination	0.62 (0.47–0.83)	0.001	0.99 (0.69–1.41)	0.96
Smoking habit	1.87 (1.41–2.48)	<0.001	1.46 (1.00–2.12)	0.048
COPD	2.05 (1.52–6.14)	0.002	1.96 (0.94–4.10)	0.07
Fibro-calcified lesions on chest radiograms	1.87 (1.36–2.56)	<0.001	1.56 (1.12–2.17)	0.009

a Odds ratios and 95% confidence intervals were derived from the logistic regression analysis.

OR, odds ratio; CI, confidence interval; COPD, chronic obstructive pulmonary disorder; TB, pulmonary tuberculosis; BCG, bacille Calmette-Guerin.

The patients were further stratified by sex and the predictors for LTBI analyzed accordingly (Table 3). In males, the independent factors associated with LTBI included smoking habit (OR 1.59, 94% CI 1.05–2.40; p = 0.027) and fibro-calcified lesions on chest radiogram (OR 1.80, 95% CI 1.17–2.78, p = 0.008). In females, the independent factors associated with LTBI included older age (OR 1.02, 95% CI 1.004–1.032; *p* = 0.009), absence of malignancy (OR 0.50, 95% CI 0.28–0.87; *p* = 0.016), and history of gastrectomy (OR 11.15, 95% CI 1.22–101.84; *p* = 0.033).

**Table 3:** Univariate and multivariate analysis of clinical factors associated with latent TB infection between sexes^a^.

	Male				Female			
	Univariate analysis		Multivariate analysis		Univariate analysis		Multivariate analysis	
	OR (95% CI)	*p* value	OR (95% CI)	*p* value	OR (95% CI)	*p* value	OR (95% CI)	*p* value
Age	1.02 (1.01–1.03)	<0.001	1.01 (1.00–1.03)	0.09	1.01 (1.00–1.03)	0.040	1.02 (1.00–1.03)	0.009
BCG vaccination	0.55 (0.38–0.79)	0.001	0.97 (0.61–1.64)	0.91	0.78 (0.51–1.21)	0.27		
Smoking habit	2.06 (1.41–3.01)	<0.001	1.59 (1.05–2.40)	0.027	0.68 (1.90–2.42)	0.55		
COPD	2.65 (1.30–5.40)	0.007	1.93 (0.91–4.09)	0.09				
Malignancy	1.26 (0.88–1.82)	0.21			0.61 (0.36–1.04)	0.07	0.50 (0.28–0.87)	0.016
Post gastrectomy	1.57 (0.54–4.60)	0.41			12.20 (1.35–110.26)	0.026	11.15 (1.22–101.84)	0.033
Autoimmune diseases	0.85 (0.42–1.70)	0.64			0.81 (0.48–1.35)	0.41		
Liver cirrhosis	1.16 (0.52–2.57)	0.72			0.19 (0.03–1.46)	0.11		
Fibro-calcified lesions on chest radiograms	2.16 (1.44–3.26)	<0.001	1.80 (1.17–2.78)	0.008	1.46 (0.88–2.43)	0.15		

a Odds ratios and 95% confidence intervals were derived from the logistic regression analysis.

OR, odds ratio; CI, confidence interval; COPD, chronic obstructive pulmonary disorder; TB, pulmonary tuberculosis; BCG, bacille Calmette-Guerin.

**Figure 3:**
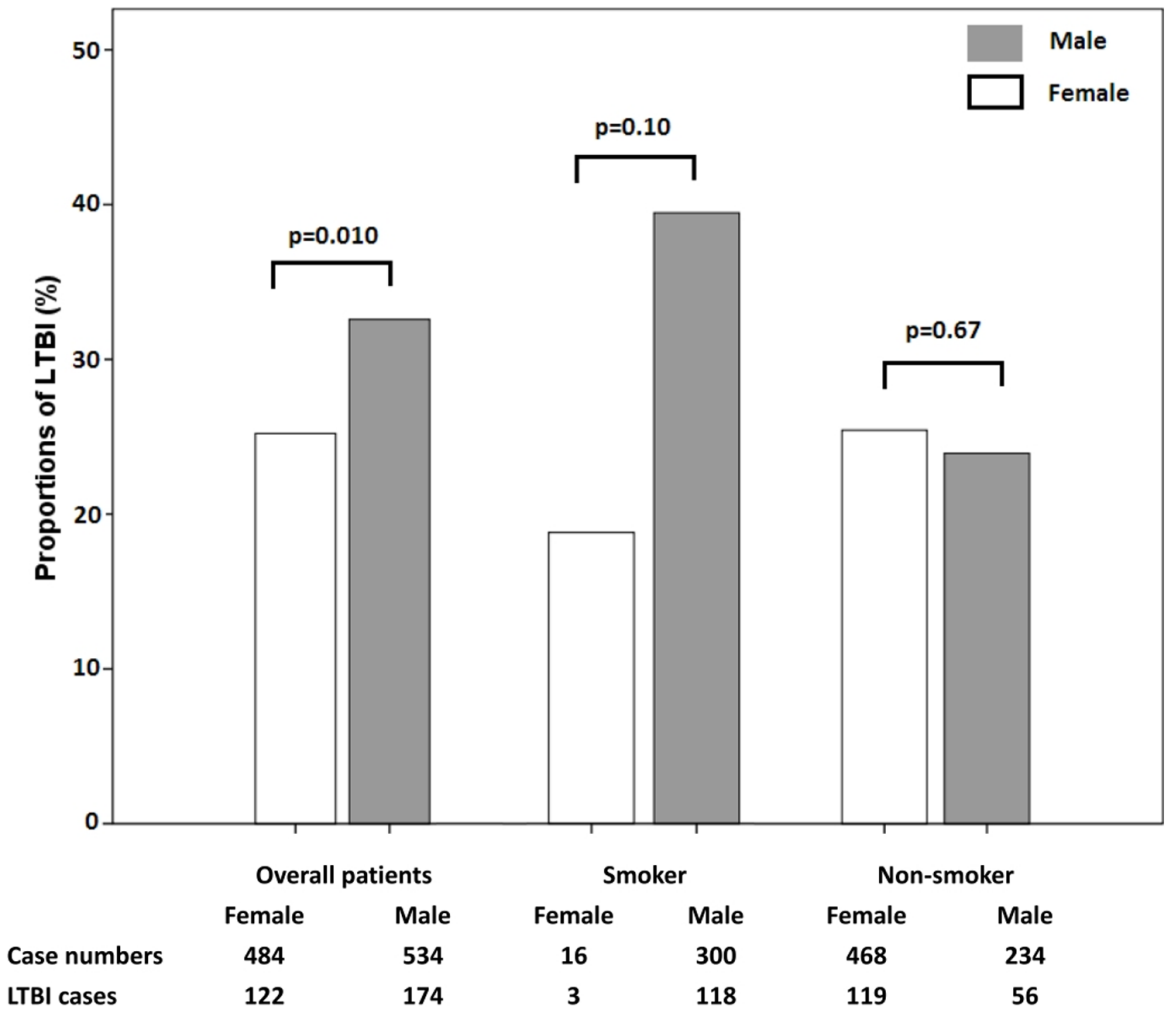
Proportions of LTBI between sexes in smokers and non-smokers.

In order to clarify the effects of smoking on LTBI between sexes, the proportion of LTBI between sexes among smokers and non-smokers were compared. Among smokers, the proportions of LTBI were 39.3% in males and 18.8% in females. Among non-smokers, the proportions were 23.9% in males and 25.4% in females. There were no statistically significant differences in the proportions of LTBI between sexes in both smokers (p = 0.10) and non-smokers (p = 0.67) (Figure 3).

## Discussion

This cross-sectional study enrolled high-risk individuals from a TB-endemic area and analyzed the disparities in terms of sex in LTBI. More than 25% of patients had LTBI. By univariate analysis, the proportion of LTBI was significantly higher in men than in women. The proportion of LTBI significantly increased with increasing age in both men and women, and the sex disparity was most prominent in the elderly populations. When stratifying the patients according to their smoking habit, the proportions of LTBI were comparable between sexes in both smokers and non-smokers. By multivariate analysis, male sex was not an independent factor associated with LTBI. Other independent factors associated with LTBI included age, smoking habit, and fibro-calcified lesions on chest radiograms.

As sex disparities in active TB are widely reported globally, several possible causes are proposed to explain the observations. The gender inequalities in cultural and social aspects are enormous in some developing countries that are also TB-endemic areas. These inequalities affect the help-seeking behaviors and reduce the access of women to health care services. The under-notification of active TB cases in females may be due to the gender bias in these areas, especially in a passive case-finding setting [12], [13], [23]. Moreover, men may have more social contact than women and thus lead to an increased risk of exposure to contagious case [24]. However, in a large population prevalence survey carried out in Bangladesh that used an active-case finding strategy, excess TB cases in males with male-to-female ratio of 3∶1 was still reported [25]. Although the authors concluded that disparities between sexes in active TB are real, several potential confounding factors like smoking, drug use, and air pollution exposure were not adjusted in their study [26], [27].

The present study analyzed the prevalence of LTBI between sexes in a TB endemic area. Since cases with LTBI were asymptomatic, this cross-sectional study was in an active case-finding setting. Socio-cultural disparities between sexes are less remarkable in Taiwan. In addition, there is high medical accessibility since 99% of the population has universal medical coverage by the National Health Insurance Program. In our study, although men have higher proportion of LTBI than women, with odds ratio of 1.43 in univariate analysis, male sex is not an independent factor associated with LTBI after adjusting for age, smoking habit, and other clinical factors. These results support previous observations that males have higher a probability of having TB infection with active disease progression. However, the results also indicate that age and smoking may play important roles in sex disparities in TB.

Because males in the enrolled population are older and more likely to have a smoking habits, the patients were further stratified by age and smoking status to evaluate their roles in sex disparities. Disparities between sexes were most prominent in the elderly population and the proportion of LTBI is comparable between sexes in patients younger than 45 years old. Such findings are comparable with those reported in Bangladesh as their female-to-male ratio also declined rapidly in patients older than 45 years old [25]. Elderly males may have more co-morbidities that lead to a higher proportion of LTBI. Prolonged smoke exposure in elderly male is also a possible factor.

Smoking is a well-established risk factor for lung disorders, including TB [28]. Some animal studies demonstrate that smoke exposure impairs pulmonary immunity and increased susceptibilities to mycobacterial infection [29], [30]. Thus, the higher smoking rate in males and longer duration of smoking in elderly males may be other important factors for sex disparities. Interestingly, the proportion of LTBI is comparable between men and women without smoking. Among smokers, the proportion of LTBI is higher in men but without statistical significance. Due to the small case number of females with a smoking habit, the impact of smoking on LTBI in females cannot be readily evaluated in the present study. Nonetheless, the findings here strongly suggest that smoke exposure plays at least a partial role in sex disparities in LTBI in Taiwan. The anti-smoking campaign and smoke cessation program are important for TB control and should be incorporated in any TB control program.

The present study has several limitations. The participants were from a referral medical center. Patients with older age and more co-morbidities were more likely to be enrolled. The study included high-risk individuals with different disease entities so the patient population was heterogeneous, which made the findings applicable to various high-risk individuals. However, it also limited our findings to be applied in general populations. Although patients with previous and current anti-TB treatment were excluded, a significant portion of the patients had fibro-calcified lesions on chest radiograms, which is suggestive of previous TB disease. The remote immune memory may confound the QFT-GIT results. Nevertheless, their impact has been adjusted in the multivariate analysis. Few cases of female smokers have been enrolled in the present study. Further studies with larger sample sizes are needed to elucidate the impact of smoking on LTBI in females. Lastly, Taiwan is a TB-endemic area with a low prevalence for HIV. Testing for HIV was not routinely conducted among the enrolled patients. This limits the applicability of the findings to countries with a low prevalence of TB- or HIV-endemic areas.

In conclusion, male high-risk individuals in Taiwan have a higher proportion of LTBI compared to females. The proportion of LTBI increases with increasing age in both men and women, and disparities in sex are most prominent in the elderly populations. Male sex is not an independent predictor for LTBI by multivariate analysis. There is also no statistically significant difference in the proportion of LTBI between sexes in both smokers and non-smokers. Other independent factors associated with LTBI include age, smoking, and fibro-calcified lesions on chest radiograms. The role of smoking in disparities in sexes in LTBI deserves further studies and highlights the importance of the anti-smoking campaign in TB control.
